# Insights Into the Role of Vitamin D as a Biomarker in Stem Cell Transplantation

**DOI:** 10.3389/fimmu.2020.00966

**Published:** 2020-06-08

**Authors:** Jose Ros Soto, Chloe Anthias, Alejandro Madrigal, John A. Snowden

**Affiliations:** ^1^Anthony Nolan Research Institute, Royal Free Hospital and University College London, London, United Kingdom; ^2^Department of Haemato-Oncology, The Royal Marsden Hospital, Sutton, United Kingdom; ^3^Department of Haematology, Sheffield Teaching Hospitals NHS Foundation Trust, Sheffield, United Kingdom

**Keywords:** supportive care, Vitamin D, hematopoietic stem cell transplantation, 25(OH)D3, post-transplant complications, graft-versus-host disease

## Abstract

Vitamin D was discovered 100 years ago and since then multiple studies have consistently proved its effect on bone health and mineral metabolism. Further research has also explored its so-called “non-classical” biological effects, encompassing immune regulation and control of cell proliferation and differentiation. Vitamin D downregulates pro-inflammatory immune cells and subsequently their cytokine production, while enhancing the anti-inflammatory subsets, thus mediating inflammation and fostering a more tolerogenic environment. Its biological action is exerted through the vitamin D receptor, a nuclear receptor that mediates gene transcription and is expressed in most cells from the innate and adaptive immunity. Owing to its immune-modulatory properties, its role in cancer pathophysiology, hematology disorders and stem cell transplantation has also been investigated. Vitamin D deficiency causes immune imbalance and cytokine dysregulation, contributing to some autoimmune diseases. In the hematopoietic stem cell transplant setting this could lead to complications such as acute and chronic graft-versus-host disease, ultimately impacting transplant outcomes. Other factors have also been linked to this, including specific polymorphisms of the vitamin D receptor in both stem cell donors and recipients. Nevertheless, studies thus far have shown conflicting results and the use of vitamin D or its receptor as biomarkers has not been validated yet, therefore there are no evidence-based consensus guidelines to guide clinicians in their day-to-day practice. To gain more insight in this topic, we have reviewed the existent literature and gathered the current evidence. This is an overview of the role of serum vitamin D and its receptor as biomarkers for clinical outcomes in patients undergoing hematopoietic stem cell transplantation. Further prospective studies with larger cohorts are warranted to validate the viability of using serum vitamin D, and its receptor, as biomarkers in potential stem cell donors and patients, to identify those at risk of post-transplant complications and enable early therapeutic interventions.

## Introduction

Vitamin D has received considerable attention in recent years due to its non-skeletal functions (1, 2), particularly immune regulation (3). Vitamin D receptor-mediated signaling promotes innate immunity and modulates adaptive immune responses (4–8). This has reinvigorated the interest in vitamin D in the field of hematopoietic stem cell transplant (HSCT) (9–14), where recipients are at high risk of vitamin D deficiency (15–20). Since this can lead to complications post-HSCT, including graft-versus-host disease (GvHD), identifying patients at risk of vitamin D deficiency is crucial to enable prompt therapeutic interventions and reduce transplant-related morbidity and mortality (9, 19).

At the beginning of the twentieth century, rickets had become a major public health issue due to its high incidence in the UK population. At the University of Sheffield, Professor Sir Edward Mellanby performed extensive research on dogs with rickets that led to the discovery of vitamin D in 1919. It was called the “*antirachitic accessory factor*,” “*antirachitic vitamin*,” or “*fat-soluble vitamin”* (as it was contained in butter and animal fat) (21, 22). In cooperation with his wife, May Mellanby, they studied puppies and found that the cod-liver oil had a fundamental role in bone calcification (23).

Professor Mellanby extrapolated his research to humans, where lower-social-class children with a diet rich in milk (included those who were breastfed), eggs, or fish had a lower incidence of rickets, better jaws and teeth compared to those from the high class, whose diets were lacking in these aliments (21).

### Vitamin D Metabolism

Vitamin D is a fat-soluble secosteroid (steroid with a “broken” ring) (8, 24) mainly synthesized in the skin (70–80%) (25). The remaining 20–30% is consumed with diet: Mushrooms, egg yolk, and oily fish (mackerel, sardines, herrings and salmon) contain high concentrations of vitamin D (8). For decades, cod liver oil has been regularly used for both the prevention and treatment of infectious diseases, such as tuberculosis (26, 27). When taken with the diet, both vitamin D^2^ and vitamin D^3^ are absorbed in the small bowels similarly to lipids and then transported to the liver through the lymphatic vessels (28).

When the solar ultraviolet light B radiation (spectrum 280–320 UVB) hits the epidermis, the 7-dehydrocholesterol (also called *pro-vitamin D*) is transformed into *pre-vitamin D*^3^ (29). Immediately after, a thermal reaction produces the isomerization of this into vitamin D^3^, or *cholecalciferol*, the inactive form of vitamin D. The higher the UVB intensity, the higher the quantity of vitamin D^3^ is synthesized. This process takes up to 3 days after the skin has been exposed to sunlight. Consecutively, the vitamin D^3^-binding protein (DBP; an alpha-1 globulin plasma carrier) bounds to vitamin D^3^ and releases it into the bloodstream (30).

The first hydroxylation is held in the liver, and the main enzyme is *25-hydroxylase* (CYP2R1) (6). The quantity of 25(OH)D^3^ or *calcidiol* hydroxylated is proportionate to the total amount of vitamin D both synthesized and ingested with the diet, thus making this the most reliable marker of vitamin D serostatus (31). This is still inactive but has a longer lifespan (between 2 and 3 weeks) than its active counterpart (32). The second hydroxylation takes place primarily in the kidney by *1*α*-hydroxylase* (CYP27B1) (6). *Calcitriol* or 1,25(OH)_2_D^3^ is the biologically active hormone (24). CYP27B1 is also found in other organs, including skin, lymph nodes, colon, central nervous system, adrenal glands, pancreas, placenta, sweat glands and the immune cells (6, 7, 33, 34). Finally, *24-hydroxylase* (CYP24A1) catabolizes 1,25(OH)_2_D^3^ into *calcitroic acid*, functionally inactive. This is excreted through the bile and subsequently the faeces, as well as the urine, avoiding toxic levels (35). This reaction occurs in cells that possess the vitamin D receptor (VDR) (1, 6, 24). Interestingly, CYP24A1 is upregulated in tumor cells to abrogate the vitamin D–related anti-tumor effects (36).

### Vitamin D Receptor (VDR)

Vitamin D acts as a ligand-inducible transcription factor binding to the VDR, a member of the nuclear hormone receptors superfamily. It is located in most of the cells in humans, including those within the immune system (7).

Vitamin D, as a lipophilic molecule, passes through the cellular membrane and binds the VDR in the nucleus. The vitamin D–VDR complex forms a heterodimer with the Retinoid X Receptor (RXR), which is subsequently bound to the Vitamin-D-Responsive Elements (specific sequences of DNA in the promoter region of the vitamin D responsive genes), controlling the transcription of these genes (32, 37). On the one hand, some genes can be upregulated by 1,25(OH)_2_D^3^ itself, including those encoding CYP24A1, leading to an increase catabolism of 1,25(OH)_2_D^3^, or CAMP, that enhances the production of cathelicidin, an antibacterial peptide. On the other hand, it downregulates genes, such as those of IL-2 and IFN-γ (interferon gamma) in T cells (7). Interestingly, VDR in osteoblast mediates between the nervous system and the bone marrow niche, promoting stem cells mobilization after G-CSF (*granulocyte colony stimulating factor*) administration (38).

### Vitamin D Function

**Figure 1:**
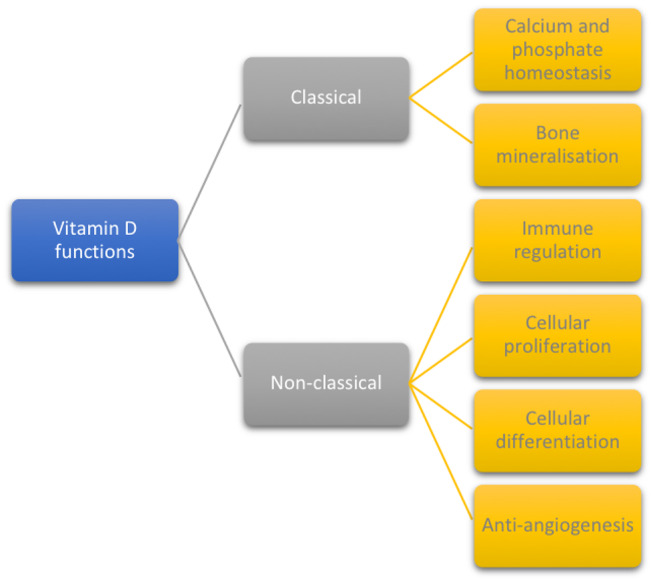
Classical and non-classical functions of vitamin D.

The biological functions of vitamin D are divided into classical (32, 39, 40) and non-classical (1, 6, 24), as displayed in Figure 1.

## Effect of Vitamin D in the Immune System

VDR is found in cells from innate (3, 41–46) and adaptive (3, 42, 47–50) immunity. Vitamin D exerts its immune-regulatory function, inhibiting the pro-inflammatory cells with a subsequently downregulation of their hallmark cytokines while enhancing the anti-inflammatory subsets, maintaining the immune tolerance (4–8). As an example, pro-inflammatory cytokines, such as TNF-α (tumor necrosis factor alpha), IL-1 and IL-6 decrease during summer months, when vitamin D reaches its peak level in blood (4, 6, 8).

Immune cells can transform 25(OH)D^3^ into its active form because they express the enzyme CYP27B1 (8, 42, 51, 52). In addition, they control the local metabolism of vitamin D self-consuming the manufactured vitamin or secreting to the adjacent cells (8, 33). However, for optimal modulation of immune responses, this system relies on the availability of systemic 25(OH)D^3^, as 1,25(OH)_2_D^3^ has a very short half life (8).

### Innate Immunity

Vitamin D targets antigen-presenting cells as follows:

In neutrophils, vitamin D contributes to tissue preservation hampering IL-1b, a pro-inflammatory cytokine synthesized by neutrophils (53). In addition, an *in vivo* study showed that 1,25(OH)_2_D^3^ acts as a differentiation agent in leukemic retinoic acid-resistant promyelocytes into mature granulocytes (54).

Moreover, a link between 1,25(OH)_2_D^3^ and early neutrophil recovery post-HSCT suggest the potential role of this vitamin in immune reconstitution (10).

The production of 1,25(OH)_2_D^3^ increases throughout the maturation of dendritic cells (DCs) due to a higher expression of CYP27B1 (8). However, 1,25(OH)_2_D^3^ keeps DCs in an immature state to preserve immune tolerance (43, 55, 56). From the DCs perspective, 1,25(OH)_2_D^3^ hampers interaction and priming of T cells inhibiting expression of receptors CD40, CD80, and CD86 in the DCs' surface (55, 56), diminishing the secretion of IL-12 and concurrently of IFN-γ (19, 33, 55–57), and suppressing DCs' migration to lymph nodes due to reduction of CCL21 and its receptor CCR7, blunting antigen presentation to T-cells (43, 44). It mainly impacts on the myeloid DCs, which interact and activate naïve T cells (57).

Vitamin D fosters macrophage maturation and enhances phagocytosis (3, 51). During infections, CYP27B1 is upregulated by viruses, cytokines, such as IFN-γ or lipoproteins from the Mycobacterium membrane, resulting in an increase of 1,25(OH)_2_D^3^ synthesis. In addition, vitamin D regulates the expression of specific endogenous antimicrobial peptides, such as cathelicidin (8, 26, 51, 58), which has also been found to possess tumoricidal activity against high-grade lymphoma cells, contributing to rituximab-mediated cytotoxicity (59). Furthermore, vitamin D downregulates the expression of MHC (major histocompatibility complex) class II on the macrophage surface, hindering T-cell activation (41) and decreasing the pool of circulating CD16^+^ monocytes and their secretion of TNF-α (60).

Natural killer cells (NK) proliferation and cytotoxic function is abrogated by 1,25(OH)_2_D^3^, inhibiting the secretion of TNF-α and IFN-γ (46, 61). In the innate NK cells, it also upregulates the secretion of IL-4 (62).

### Adaptive Immunity

VDR is also upregulated in activated B lymphocytes (63), inhibiting the synthesis of immunoglobulins (6, 47) and decreasing B cell proliferation and differentiation into plasma cells (64). Moreover the expression of CYP24A1 enables B cells to degrade 1,25(OH)_2_D^3^ into calcitroic acid and subsequently to eliminate it (42).

Vitamin D blunts inflammation and alloreactivity because it reduces the pool of activated T lymphocytes (50) and the production of TNF-α, as shown in a study carried out in HSCT patients (64). VDR is upregulated in the activated T cells as well as in the naïve and early memory subsets, acting as a subrogate marker of T-cell activation (50, 65). To ensure sufficient supply of 1,25(OH)_2_D^3^ is provided to the neighboring cells (8, 62), CYP27B1 is upregulated, as well as 24α-hydroxylase to avoid an overproduction of this vitamin (52).

In CD4^+^, on the one hand, 1,25(OH)_2_D^3^ downregulates the production of IL-2 and IFN-γ by Th1 (52, 64–66) and impairs IL-17 secretion by Th17 (62, 67). On the other hand, it helps expanding the pool of Th2 cells, with a subsequent upregulation of their landmark cytokines. One of them, IL-4, also triggers 24α-hydroxylase to prevent supra-physiological levels (51, 67). Part of the immune-modulatory effect of 1,25(OH)_2_D^3^ is due to the enhancement of the IL-6 secretion, which abrogates the Th1 cells, skewed in favor of the anti-inflammatory and pro-tolerogenic Th2 subset (55).

Some studies have found contradictory results on the effect of vitamin D in CD8^+^ T-cell proliferation, thus currently, no conclusions can be drawn (49, 64, 68).

Despite controversy in this matter (52, 68), preclinical studies have shown that 1,25(OH)_2_D^3^ triggers secretion of IL-10 by CD4^+^ T cells (69) and TGF-β (transforming growth factor beta) by DCs (55, 57, 67, 70), which ultimately enhance the recruitment of Foxp3^+^ CD25^+^ regulatory T cells (Treg) (59, 66). These CD4^+^ lymphocyte subset impairs the expansion of alloreactive donor T cells in GvHD-target tissues and subsequently the synthesis of their pro-inflammatory cytokines, including IL-2 (71). Alongside this, 1,25(OH)_2_D^3^ downregulates the expression of skin and gut-homing molecules (cutaneous lymphocyte-associated antigen and chemokine receptor CCR9, respectively) in the T cell surface, with a subsequent impairment in T cell trafficking (52, 68). This contributes to abrogate GvHD and foster a more tolerogenic immune environment (62, 70, 72).

Moreover, a preclinical study postulated that a population of IL-10-secretor B cells could act as regulatory immune cells, but data is limited so further research is needed (52).

## Vitamin D Deficiency

Currently, vitamin D deficiency is considered a pandemic disease (73). Although its prevalence in higher latitudes is well known, it can also affect individuals living in areas closer to the Ecuador (74). It can also affect individuals living in areas closer to the Ecuador (25, 74–76).

Many factors have been identified to contribute to it: age (77), low sunlight exposure (25), skin pigmentation, obesity and decrease of cutaneous synthesis of vitamin D. HSCT recipients can also suffer from malnourishment (31), malabsorption, or gut GvHD (35), which can have a detrimental impact on absorption of vitamin D-enriched aliments. In addition, vitamin D metabolism can be altered by immunosuppression (35, 78–80) or as a consequence of kidney (35) or liver (79) impairment. Moreover, some genetic polymorphisms in genes related to the vitamin D metabolism have been identified in individuals at risk of vitamin D insufficiency (81).

The half life of the inactive metabolite 25(OH)D^3^ has been estimated to be between 2 and 3 weeks. It identifies individual adequacy or insufficiency, making it the most useful marker of the vitamin D body stores (32).

For over a century, most of the research performed regarding the vitamin D has been looking into its effect on bone health (23). Thus it is not surprising that the cut-off established for vitamin D deficiency has been based on the optimal serum levels of 25(OH)D^3^ required to prevent bone loose while maintaining calcium homeostasis (79). Nevertheless, little is known about the levels needed to enhance immune-regulation and forestall complications following HSCT (4, 6, 8), and so a threshold that can be applied into the HSCT setting has not been validated yet (9, 82–84).

In the general population, studies regarding this have shown remarkable discrepancies: whereas the *Institute of Medicine* advocates for a cut-off of 30 nmol/L (12 ng/mL) (24), *NICE* guidelines and the *Endocrine Society Task Force on Vitamin D* established it below 25 nmol/L (10 ng/mL) (78, 85), and even one report has set it below 50 nmol/L (20 ng/mL) (86). Therefore, it is not possible to suggest a cut-off that defines vitamin D deficiency in recipients of HSCT based on the evidence published so far.

Moreover, the non-skeletal functions of vitamin D have reinvigorated its interest as potential modulator in a broad spectrum of diseases and therapeutical procedures, as follows:

### Autoimmune Diseases

Despite some clinical studies focused on the role of vitamin D deficiency have revealed its contribution to the pathophysiology of some autoimmune diseases, including multiple sclerosis, systemic sclerosis, rheumatoid arthritis, insulin-dependent diabetes and systemic lupus erythematous (4, 7, 45, 87), others could not reproduce these results (88, 89).

### Asthma

Interestingly, studies performed in patients with asthma showed that patients with lower serum levels of vitamin D were less responsive to steroids than those with higher levels. The reason for this is the impaired steroid induction of IL-10 secretion by CD4^+^ T cells, leading to a poor recruitment of Tregs. However, it can be restored with vitamin D supplementation: Due to its immunomodulatory properties, vitamin D enhances the secretion of IL-10 by CD4^+^ T cells, increasing the pool of both population of circulating Tregs (Foxp3^+^ and IL-10 Tregs) *in vitro* and contributing to the control of the disease, as seen in clinical studies (66, 70, 72, 90).

### Infectious Diseases

Vitamin D has been used as a biomarker for critically ill patients with sepsis, whom levels of 25(OH)D^3^ were lower than those from patients also admitted in Intensive Care Unit but without sepsis (58). However, despite the evidence found in a few preclinical studies about the effect of 1,25(OH)_2_D^3^ fostering macrophage activity against Mycobacterium tuberculosis (49) or downregulating cytokine production during viral infections (91), data regarding infections is still controversial, including studies in HSCT patients (11, 49, 92, 93). Similarly, in a clinical study where vitamin D supplementation was given as adjunctive therapy to vaccinations, it did not show any clinical relevance (91).

### Cancer

*In vitro* studies have shown that 1,25(OH)_2_D^3^ inhibits cellular proliferation (downregulating BCL-2 expression and telomerase activity) and angiogenesis (inhibiting *VEGF*, vascular endothelial growth factor), and acts as a pro-apoptotic and differentiation-inducing agent in a range of malignant cells (1, 6, 36, 54, 94–96) because these cells possess VDR (97). In clinical studies, vitamin D serostatus has been linked to solid tumors, including melanoma (98–100), breast (6, 101, 102), colon (6), prostate (102), and lung cancer (103). Furthermore, this anti-tumor effect has also been investigated in hematology disorders, such as myelodysplastic syndrome (96), myeloid leukemias (95, 96), and multiple myeloma (104). In some reports, higher levels of 1,25(OH)_2_D^3^ have been found to impact favorably in survival (97, 99, 103, 105). However, there has been some discrepancy in lymphoid malignancies, as a few studies found a positive impact of 1,25(OH)_2_D^3^ in outcomes (95, 97, 106) whereas others did not (107, 108). Moreover, Hansson et al. showed that patients with malignant hematological disorders and vitamin D deficiency before transplantation could have higher relapse rate compared to those patients whom levels were higher (10). Supporting this, another paper mentioned similar results in patients with myeloid malignancies (109), whereas another failed to reproduce the same results (64).

### Solid Organ Transplantation

Vitamin D deficiency is highly prevalent in heart and liver transplant recipients, predominantly in the latter because end-stage liver failure alters vitamin D^3^ first hydroxylation (110). Furthermore, nearly 50% of lung transplant recipients are vitamin D deficient, as reported by one single center study. In this population, low levels of 25(OH)D^3^ were linked to worse pulmonary function tests and higher graft rejection (111). Moreover, recent reviews have reported how chronic kidney disease and kidney transplant can aggravate hypovitaminosis D and how patients with lower 25(OH)D^3^ serum levels were more likely to suffer from secondary tumors and graft rejection, leading to a poorer survival after transplantation (104, 112). In this context, vitamin D supplementation can play a reno-protective role (113).

## Impact of Vitamin D in HSCT

Vitamin D deficiency can contribute to the imbalance of immune homeostasis, shifting from a tolerogenic to a pro-inflammatory status (89, 113). In the allogeneic HSCT, this can have an impact on complications post-transplantation, and potentially on survival outcomes (9, 11, 114, 115).

### Immune Reconstitution Post-HSCT (Figure 2)

Early immune recovery is characterized by neutrophil engraftment. At this stage, 1,25(OH)_2_D^3^ may enhance neutrophil recovery, as shown in a pediatric study where patients with higher levels of 25(OH)D^3^ had a higher neutrophil count at the time of engraftment (10). Nevertheless, other studies have failed to prove this (11, 82). Moreover, two reports suggested the contribution of donors' VDR genotype in the late immune reconstitution of T cells (116, 117), but data is still limited to draw any conclusion.

**Figure 2:**
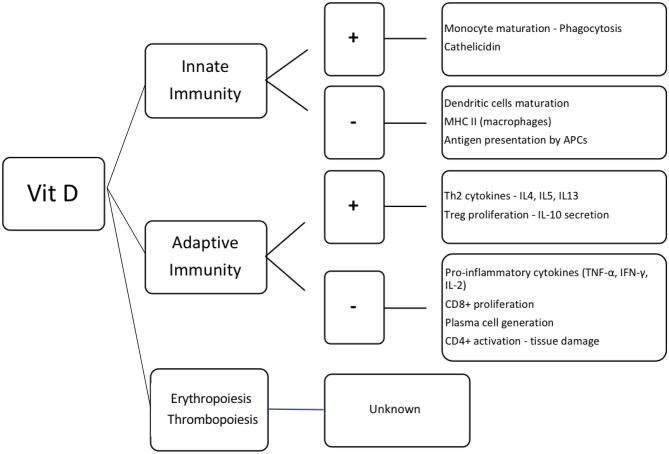
Effect of vitamin D in the hematopoietic cells. Vit D, vitamin D; +, activation; –, inhibition; MHC II, major histocompatibility complex class II; APCs, antigen presenting cells; Th2, T helper lymphocytes 2; IL, interleukin; Treg, regulatory T cells; TNF-α, tumor necrosis factor α; IFN-γ, interferon γ; CD8^+^ and CD4^+^, T lymphocytes CD8^+^ and CD4^+^, respectively.

Beyond its immune-modulatory properties, 1,25(OH)_2_D^3^ stimulates proliferation and differentiation of CD34^+^ hematopoietic stem cells (118–120). It also inhibits secretion of pro-inflammatory cytokines, such as IL-6 and subsequently hepcidin production, resulting in stimulation of erythropoiesis (121–123). However, little is known of its effect on thrombopoiesis (124).

### Graft-versus-Host Disease

GvHD is a major complication following allogeneic HSCT and one of its main causes of death (125). Clinical studies have suggested the link between vitamin D deficiency and GvHD (9, 69, 82, 84). Acute GvHD (aGvHD) pathophysiology is characterized by a strong inflammatory reaction (126), while chronic GvHD (cGvHD) shares features of autoimmunity (127, 128). Vitamin D deficiency causes immune imbalance and cytokine dysregulation, with expansion of autoreactive T cells, enhancing the response of these immunologically competent cells against host antigens, and blunting vitamin D–mediated immune homeostasis (113, 129).

Surprisingly, vitamin A has also been suggested to be involved in GvHD pathogenesis (130), but its potential mechanistic effects of on GvHD are yet to be properly characterized (131).

Three clinical studies have linked 1,25(OH)_2_D^3^ serostatus and acute GvHD (aGvHD): Urbain et al. demonstrated that patients with moderate to severe aGvHD had lower levels of 25(OH)D^3^ after HSCT (69). Kreutz et al. correlated a higher grade of aGvHD with vitamin D deficiency (82). Finally, Ganetsky et al. found that those patients with vitamin D deficiency had an increased risk of grade II–IV skin GvHD (132). Nevertheless, these results could not be reproduced in other studies (10, 69, 84, 115).

Glotzbecker et al. reported that patients with lower levels of 25(OH)D^3^ prior to HSCT had a higher cumulative incidence of Chronic GvHD (cGvHD) and extensive cGvHD compared to those with higher levels (84). Supporting this, another clinical study showed that cGvHD developed in patients with lower 25(OH)D^3^ serum levels at transplantation (9). In contrast, other clinical studies failed to find any correlation between vitamin D serostatus and cGvHD (10, 115, 132).

Currently there is controversy in the evidence of the impact of vitamin D deficiency within the GvHD pathophysiology. Therefore, further studies with larger sample size to confirm this are warranted.

### Resistance To Steroids in GvHD

More than 50% of patients treated with steroids for GvHD are resistant to this immunosuppressive treatment (133–135). The cause for this remains unknown but there is strong evidence linking this to a poorer chance of survival in these patients (136).

In the field of asthma, recent studies have linked vitamin D serostatus with steroid resistance: lower levels of serum 25(OH)D^3^ were associated with poorer steroid response (72, 137). Nevertheless, treatment with 1,25(OH)_2_D^3^ overcame this, resulting in clinical improvement of asthma severity (66, 70, 72, 90). Vitamin D replacement enhanced the expansion of Treg due to the increased secretion of IL-10 by CD4^+^ T cells, previously unresponsive to steroids (70, 72, 138).

In the steroid-resistant GvHD setting, one preclinical study suggested that synergism between vitamin D supplementation and steroids could abrogate the monocyte-induced release of pro-inflammatory cytokines and therefore mitigate the tissue damage by GvHD (139).

These findings serve as a rationale for treating or preventing vitamin D deficiency by upholding normal levels of vitamin D in order to enhance the immunosuppressive effect. Since vitamin D may overcome the resistance to immunosuppression in GvHD, further research in this field is needed to confirm this hypothesis and potentially to reduce the morbidity and mortality associated to this disease.

### Outcomes Post-HSCT (Table 1)

As previously described, vitamin D has an immune-modulatory role, and it may protect against infections and blunt tissue damage on the course of HSCT (54, 91). Owing to this, recent studies have tried to elucidate its role in outcomes following allogeneic HSCT, with conflicting results: A prospective study performed in pediatric patients revealed that vitamin D deficiency post-HSCT was associated with a lower overall survival (OS) (114), as seen in other studies evaluating OS at different time points (9, 11, 115). Nevertheless, further research could not prove the link between vitamin D serostatus and progression-free survival (84, 115), 2-years disease-free survival (9), or OS (84, 132, 141), thus no definitive conclusions can be drawn from them.

**Table 1:** Observational studies correlating vitamin D status with outcome post-HSCT*.

**References**	**Study design**	Age population (N**)	**VDD threshold**	**VDD** **pre-HSCT**	**VDD post-HSCT**	**GvHD**	**Outcomes**
Kreutz et al. (82)	NR	NR (48)	<25 nmol/L	Serum 25(OH)D^3^: 36.4 (±2.2 nmol/L)	Serum 25(OH)D^3^: 27.8 (±1.3 nmol/L)	Lower levels of 25(OH)D^3^ in grade III and IV aGvHD (*P* = 0.031)***	NR
Joseph et al. (18)	Prospective	Adult (72)	<20 ng/mL	70%	58%	NR	NR
Sproat (20)	Retrospective	Adult (58)	<20 pg/mL	NR	59%	NR	NR Comment: 21% of patients on VD supplements
Urbain et al. (69)	Prospective	Adult (102)	<10 ng/mL	23.5%	NR	Weak association in patients with lower levels of 25(OH)D^3^ on day + 100 and aGvHD (*P* = 0.066)	NR
Glotzbecker et al. (84)	Retrospective	Adult (53)	<25 ng/mL	60%	NR	No significant differences in aGvHD 2-years CI of cGvHD: 63.8% in VDD patients compared to 23.8% in sufficient VD patients (*P* = 0.02) Extensive cGvHD at 2-years was 54.5% in VDD patients compared to 14.3% in sufficient VD patients (*P* = 0.009)	No impact on OS (*P* = 0.57) nor PFS (*P* = 0.61)
Simmons et al. (77)	Prospective	Pediatric (22)	<15 ng/mL	27%	NR	NR	NR
Hansson et al. (10)	Prospective	Pediatric (123)	<50 nmol/L	69%	NR	More frequent in patients with sufficient VD compared to VDD patient (47 vs. 30%; *P* = 0.05) No significant differences in cGvHD	Lower OS in patients with malignancies and VDD compared to those VD sufficient (50 vs. 87%; *P* = 0.01) Relapse rate higher in VDD compared to normal VD levels (33 vs. 4%) (*P* = 0.03) No significant association with CMV and EBV reactivation
Wallace et al. (12)	Prospective	Pediatric (135)	<20 ng/mL	NR	23%	No significant differences in a/cGvHD	Lower OS in VDD*** (*P* = 0.044) 16% patients on VD supplements pre-HSCT
Von Bahr et al. (9)	Retrospective	Adult (166)	<25 nmol/L	11%	NR	No association between 25(OH)D^3^ serum levels and aGvHD Strong correlation of cGvHD with 25(OH)D^3^ serostatus (RR 2.66)	Decreased 2-years OS in VDD patients compared to sufficient VD patients (63 vs. 76%) (*P* = 0.03) VDD pre HSCT was associated with increased CMV disease (*P= 0.005*) No association with 2-years DFS
Florenzano et al. (19)	Retrospective (36% autologous and 64% allogeneic HSCT)	Adult (46)	<20 ng/mL	17%	85%	NR	NR Comment: 53% of patients on VD supplements (but not an interventional study)
Myers et al. (140)	Retrospective	Pediatric (64)	<30 ng/mL	NR	73%	NR	NR

VD, vitamin D (25(OH)D^3^); VDD, vitamin D deficiency; OS, overall survival; aGvHD, acute graft-vs.-host disease; cGvHD, chronic graft-vs.-host disease; NR, not reported; RR, relative risk; DFS, disease-free survival; PFS, progression-free survival; CI, cumulative incidence; CMV, cytomegalovirus; EBV, Epstein-Barr virus.

*Studies performed in allogeneic HSCT unless otherwise specified.

**N, number of participants tested for 25(OH)D^3^.

***Number patients affected NR.

### VDR as Biomarker in HSCT

The VDR gene is located in chromosome 12 (142). Specific single nucleotide polymorphism (SNPs) in this gene, such as *Fok*l FF and *Apa*I aa reflect upregulation of the VDR activity, whereas *Apa*I AA downregulates it, impacting on the activity of Th1 and Th2 on the early immune reconstitution following HSCT (116, 143). Furthermore, other SNPs in the VDR and CYP2R1 genes can increase the concentration of 25(OH)D^3^ in serum following supplementation with vitamin D (144, 145).

The association of VDR gene polymorphisms with major clinical outcomes following HSCT has been investigated in different studies with inconclusive results (116, 143, 146–150). Therefore, further research in this field is warranted with larger study samples, including more recipients of different donor types (unrelated, haploidentical).

## Management of Vitamin D Deficiency in HSCT

A recent survey performed across European HSCT centers described discrepancies in monitoring and replacement of vitamin D deficiency in HSCT patients: Half of the centers requested vitamin D prior to transplantation whereas nearly 80% followed this practice after it. The main reason for this could be that guidelines only recommend measuring vitamin D in the post-HSCT setting, aiming to prevent bone loss and fractures. Moreover, the cut-off for serum 25(OH)D^3^ to commence on vitamin D therapy varied across centers depending on geographical location, ranging from 25 to 100 nmol/L (14). Awareness of the immune-regulatory properties of vitamin D and its potential impact on immune reconstitution post-HSCT and GvHD were acknowledged by a minority of centers (24 and 17%, respectively), being the main reason to commence on vitamin D therapy the maintenance of calcium metabolism and bone health (62%). Since the optimal dose of vitamin D replacement has not been standardized yet in the HSCT population and this differs between pediatric and adult population (ranging from 1,000 IU per day to 600,000 IU per week) (11, 64, 141, 151–155), dosage prescribed by HSCT clinicians varied greatly across centers (14).

In summary, these findings reflect the lack of consensus in this topic within the HSCT community, so recommendations were provided to standardize criteria and harmonize the management of the aforementioned deficiency, encouraging monitoring serum 25(OH)D^3^ prior and after HSCT, and commence on replacement therapy if clinically indicated. Nevertheless, no conclusions were reached regarding the ideal threshold for vitamin D deficiency due to the lack of robust studies including HSCT patients (14). Different studies have used different cut-offs, which can mislead clinicians when implementing the management of vitamin D deficiency in their day-to-day clinical practice. Therefore, clinical outcomes may differ among studies and this can complicate the use of serum 25(OH)D^3^ as a biomarker in the HSCT landscape. Since this is the only survey performed in the allogeneic HSCT landscape and the recommendations provided are based on up-to-date clinical evidence, it seems reasonable to follow them.

## Conclusions

Vitamin D is a potent regulator of immune responses with impact in HSCT (9–13). Nevertheless, there are no clinical guidelines focusing on vitamin D status and its optimal levels required for prevention of post-transplant complications and enhancement of the immunosuppressive therapy. As a consequence, monitoring vitamin D can be easily neglected in the management of these complex patients.

The high incidence of vitamin D deficiency in allogeneic HSCT patients, alongside the current controversy (9, 11, 84, 114, 115, 132, 141), emphasizes the need for further studies on the impact of vitamin D deficiency and VDR gene polymorphisms on clinical outcomes to define its role as a biomarker in this setting.

Vitamin D deficiency may be the first potential easily modifiable host factor associated with post-allogeneic HSCT outcomes, thus identifying patients at high risk and optimizing its management to enable prompt therapeutic intervention is encouraged.

## Author Contributions

JSo lead the manuscript writing and CA, AM, and JSn contributed to it. All authors accepted the final version of the manuscript.

## Conflict of Interest

The authors declare that the research was conducted in the absence of any commercial or financial relationships that could be construed as a potential conflict of interest.
